# Coverage versus response time objectives in ambulance location

**DOI:** 10.1186/s12942-021-00285-x

**Published:** 2021-07-02

**Authors:** Ľudmila Jánošíková, Peter Jankovič, Marek Kvet, Frederika Zajacová

**Affiliations:** Faculty of Management Science and Informatics, University of Žilina, Univerzitná 1, 010 26 Žilina, Slovak Republic

**Keywords:** Emergency medical service, Ambulance location, Computer simulation, Response time, Coverage

## Abstract

**Background:**

This paper deals with the location of emergency medical stations where ambulances waiting to be dispatched are parked. The literature reports a lot of mathematical programming models used to optimize station locations. Most studies evaluate the models only analytically applying the same simplifying assumptions that were used in the modelling phase. In addition, they concentrate on systems operating one type of emergency units in homogeneous urban areas. The goal of our study is to identify which optimization criterion the emergency medical service (EMS) outcomes benefit from the most and which model should be used to design tiered systems in large urban–rural areas.

**Methods:**

A bi-criteria mathematical programming model is proposed. The criteria include the accessibility of high-priority patients within a short time limit and average response time to all patients. This model is being compared to the *p*-median model with a single response time objective and to a hierarchical *pq*-median model that considers two different vehicle types. A detailed computer simulation model is used to evaluate the solutions. The methodology is verified in the conditions of the Slovak Republic using real historical data on 149,474 ambulance trips performed in 2015.

**Results:**

All mathematical models improve EMS performance by relocating some stations compared to the current distribution. The best results are achieved by the hierarchical median-type model. The average response time is reduced by 58 s, the number of calls responded to within 15 min is increased by 5% and the number of high-priority calls responded to within 8 min by 6%.

**Conclusions:**

The EMS systems operating in heterogeneous areas should be designed to minimize response times, and not to maximize the number of calls served within a given time limit.

## Background

Emergency medical service (EMS) is an inseparable component of health care systems in many countries, from all income groups and regions of the world [1, 2]. Its main role is to provide first medical aid to patients in emergency situations. The organization of the EMS system substantially affects patients’ chances of survival and recovery. Therefore planning EMS at all levels (strategic, tactical and operational) represents a challenging problem that is still topical in the constantly changing socio-economic environment.

In the past two decades, an increasing demand for EMS service worldwide has been reported. Population ageing has been identified as the key factor of this phenomenon [3, 4]. Elderly people suffer from chronic diseases and mental or physical dysfunctions. They are subject to the risk of sudden worsening of their medical conditions and injuries caused by falls. Also the risk of life-threatening emergency events, such as the stroke, severe respiratory difficulties, and cardiac arrest, increases with age. As a result, elderly people require EMS at a higher rate than younger people do. Although the elderly do not constitute a large part of the whole population, their share in EMS demand is significant. For example, Veser et al. [5] analyse the situation in Bavaria, which is the largest German federal state. In 2012 people aged 75 years and over constituted about 9% of the total population but accounted for 33% of all emergency cases. Lowthian et al. [3] state that in 2008 the proportion of Melbourne’s population aged 85 years and over was 1.6% but the proportion of emergency transportations accounted for by this group was 13.6%.

The available census and EMS data show that the Slovak Republic follows this trend. The demographic trend elicits the need for changes in the EMS infrastructure so that the EMS system can operate better—save more lives, reduce permanent disablement, and improve the outcome of patients. The responsiveness of the system could be improved by better distribution of the stations so that they are closer to the locations where emergencies may occur. The discussion about the need for system reorganization due to demographic changes has started also in other countries, for example in Slovenia [4].

In this paper we focus on locating ambulance stations. The purpose of this work is to identify the best strategy for optimization of EMS infrastructure in a large-scale urban–rural area.

In the following literature review we focus on successful location models in EMS. Special attention is paid to the models dealing with different categories of patients and multi-objective models. Moreover, computer simulation in EMS infrastructure optimization is reviewed.

Optimization problems arising at emergency care pathway are surveyed in Aringhieri et al. [6]. Regarding EMS location problems, the authors focus on the models incorporating equity and uncertainty. Valuable for our research is especially Sect. 6 of the paper where the authors point out that simplifying assumptions are unavoidable in optimization and computer simulation can help to assess the performance of the planned system in practice.

A survey on recent research in healthcare facility location is supplied by Ahmadi-Javid et al. [7]. The study reveals that the maximal covering location problem (MCLP) is widely used to study location of emergency facilities. The problem allows for numerous variations and extensions, the most popular of which is the maximum expected coverage location problem (MEXCLP). The MEXCLP seeks to maximize the expected covered demand supposing an ambulance being busy with a certain probability and operating independently from other ambulances.

McLay [8] enhances the MEXCLP considering two different types of emergency vehicles and three patient classes. Calls are classified as Priority 1, 2, 3, where Priority 1 calls are life-threatening, Priority 2 calls may be life-threatening and Priority 3 calls are not life-threatening. The objective is to maximize the total number of expected Priority 1 calls responded to within a specified amount of time. The probabilities of vehicles being busy are the same for all candidate locations and are calculated by the hypercube queuing model. Knight et al. [9] deal with multiple classes of heterogeneous patients. Patients differ by medical conditions, so they have different urgency levels. The authors use the maximal expected survival location model with a different survival function for each patient class. The objective is to maximize the overall expected survival probability across all patient types. Leknes et al. [10] modify the maximal expected survival location model by Knight et al. [9]. The service time depends on the distance from a station to a demand zone, the distance from the scene to a hospital, the drop-off time and the probability of the transportation to a hospital. This way the model reflects the heterogeneity of the demand zones in the solved region. Three severity levels of calls are applied.

The models maximizing the overall expected survival probability across all patient types [9, 10] are in fact multi-objective models, where individual objectives for each patient class are combined into a single objective using the scalarization method. The main drawback of this method is how to set weights of individual objectives to make the model produce good results acceptable in practice. Another approach to cope with multiple objectives is goal programming. Alsalloum and Rand [11] optimize locations of a pre-defined number of ambulances. The objective function consists of two goals. The first is to maximize the expected coverage, and the second is to reduce the spare capacities of located ambulances. Goal programming requires a careful setting of objective targets, which is a difficult task especially if the model involves a large number of uncertain parameters. Inappropriate targets may lead to non-optimal solutions.

Aboueljinane et al. [12] supply an overview of the literature on simulation models applied to emergency medical service operations. The review covers the time period from 1969 to 2013. Computer simulation is identified as a useful tool for the analysis and improvement of EMS since it allows us to model the system in a high degree of detail that is not possible when using other methods such as mathematical programming or queuing theory. Most simulation studies support decisions on the base stations to open and the number of ambulances to assign to each open station. Aringhieri et al. [13] compare the current station locations in Milan (Italy) with locations proposed by the capacitated version of the location set covering model. Several scenarios with different ambulance speeds, the number of ambulances and dispatch protocols were evaluated by simulation. A trace-driven simulation approach was used, which means the model accepts a stream of actual call data as input. In contrast to self-driven simulation models, trace-driven simulation does not need the estimation of probability models describing the time and spatial distribution of calls and duration of service times. On the other hand, it has some shortcomings [14]: this approach requires a large amount of historical data; one has to handle erroneous records in the database of interventions; the existing data do not represent the future, so the simulation model cannot be used for mid-term and long-term planning when the demand volume will increase.

Zaffar et al. [15] recently evaluated three different deployment strategies by a trace-driven simulation model using Mecklenburg County (US) EMS data. The simulation model is not very realistic since it uses constant values for ambulances’ speed, on-scene and drop-off times. The travel times are calculated using the Manhattan distance. Ünlüyurt and Tunçer [16] compare four coverage-based models for station location and ambulance allocation via discrete event simulation model. The experiments include a case study of Istanbul and randomly generated instances. Also this simulation model uses constant travel speed and drop-off times. Every patient is supposed to be transported to a hospital. Moreover, ambulances cannot be dispatched to another call while they return to their original station.

We conclude this literature review by considering a recursive optimization-simulation approach to the ambulance location and dispatching problem [17]. The method iterates through two steps. First, an optimal location of ambulances and dispatching strategy is proposed by mathematical programming using an initial estimation of ambulances’ busy fraction. The model is a variant of the MEXCLP. Then the system with optimal infrastructure is assessed by computer simulation resulting in an updated busy fraction (equal for all ambulances) that inputs the mathematical model in the following iteration. The process is repeated until convergence is achieved. Convergence is measured by busy fraction and the location vector, respectively. The most inspiring issue for our research is the conclusion of the paper where Lanzarone et al. emphasise the necessity of using heterogeneous busy fractions especially in large case studies.

From the presented literature review one can make the following conclusions:

The research made so far has not answered the question which optimization criterion is the best proxy for health outcomes and which model should be used for designing EMS systems in a mixed urban–rural territory. Most studies do not compare different models mutually, and if they do, their comparison is based on the prescriptive model and suffers from the same simplifying assumptions that were used in the modelling phase. As Aringhieri et al. [6] emphasize, the best way of the assessment of the validity of alternative approaches is computer simulation. The simulation models published in the literature oversimplify the real operation due to the lack of operational data or for the sake of shorter computer processing time. Some common simplifications were mentioned with the references. However, location decisions are of strategic nature with long-term consequences and are associated with considerable investment costs. So, it is worth spending extra time on a careful assessment of the proposed infrastructural changes. In our opinion, the simulation model should be as realistic as possible. It should accurately capture all sub-processes of the service. The parameters of the model should be derived from real operation of the system. Of course, better model requires more computing time, but computing time does not matter in strategic planning, the outcome of the approach is more important. A similar conclusion is derived in [17].

The goal of our study is to identify the best suitable optimization criteria and corresponding mathematical programming model for designing an EMS infrastructure in a mixed urban–rural area. We do not consider investment costs associated with the redeployment of the stations. They are not extremely high because the ambulance can be housed in a fabricated building. Rather we use such optimization criteria that reflect the main goal of the EMS system—to save as many people as possible. Since this output cannot be measured when designing the system, surrogate optimization criteria are formulated instead. We concentrate on the most common criteria—response time and coverage. We aim at the validation of the modelling approaches with a detailed computer simulation model that precisely imitates the behaviour of all entities included in the system (patients, dispatchers, and ambulances).

## Materials and methods

### Description of the region of interest

In the Slovak Republic, the EMS system is centralized and managed by the National Dispatch Center for EMS. The system consists of: (1) regional dispatch centres; their main role is to receive and evaluate emergency calls and dispatch appropriate rescue units; and (2) rescue units (i.e. ambulances staffed by rescue teams) that aim at providing adequate medical care to patients. The present organization of EMS in Slovakia was established by a series of laws in 2004. Thereafter, in 2010 the regulations of the Ministry of Health defined the amount and locations of ambulance base stations across the country. The intention for the distribution of the stations was to be able to reach 95% of patients within 15 min or less after the emergency call, regardless the patient’s condition or the character of the area (urban or rural). According to the regulations, 273 stations are deployed in 211 towns and villages. Larger towns have multiple stations.

The regulations define just the town where a station should be, not its precise geographical location. A provider who gets the license to operate a given station chooses a suitable building, and so determines its address. The providers are public or private institutions. The jurisdictions of the providers are not restricted, they may operate across the whole country. The study in this paper is related to the positions of the stations in 2017, when EMS was provided by 12 agencies, Falck Záchranná, a.s. being the largest with 107 stations.

The Slovak system works in a Franco-German style, where the ambulance crew is qualified to provide on-site medical care. There are two types of ambulances. Most of them provide basic life support (BLS; Slovak abbreviation RZP) and have only a paramedic and a rescue driver on board. About one third of ambulances are well-equipped advanced life support units (ALS; Slovak abbreviation RLP). An ALS crew consists of an emergency physician, a paramedic and a driver. The staff is capable of performing additional life-saving procedures, e.g. inserting breathing tubes. The closest available ambulance to the emergency site is always dispatched regardless of its type. If it is a BLS ambulance and the incident is life-threatening, then the closest available ALS ambulance is dispatched concurrently. The rationale is that any medical treatment is better than waiting without a professional intervention for the arrival of a doctor. In 2017, total of 521,164 trips were performed by one or the other type of ambulance [18].

In this paper we focus on the relocation of the current stations. We do not want to change the number of stations because adding stations would be unacceptable due to economic reasons and closing some stations would worsen the accessibility of urgent health care. Our aim is to relocate some existing stations to other potential locations hoping that the new distribution will shorten response times.

### Modelling demand

The first task in optimization of the station locations is to define the demand zones where potential patients live. We decided to identify the demand zones with the territorial units used in the census for two reasons. The first one is that we face an emergency system whose infrastructure is spread over a large-scale area (specifically, the whole state territory) populated by millions of people (population of Slovakia in 2020 was 5,459,781). Inhabitants, i.e. potential patients, have to be aggregated in a limited number of units, so that the resulting location model can be solved by common computational resources with limited memory and in an acceptable amount of processing time. The division of the country into smaller demand zones (e.g. by a rectangular grid) would result in an intractable location problem due to a huge volume of input data and an enormous number of variables. Thus our demand zones correspond to villages and towns. The two largest cities (the capital Bratislava with 440,948 inhabitants and Košice with 238,138 inhabitants) are administratively divided into boroughs (17 boroughs in Bratislava and 22 boroughs in Košice) that are regarded as separate demand zones.

The second reason for regarding census units as demand zones is the estimation of the number of calls arising in every demand zone. The demand in particular zones can be estimated in several ways: from real data on EMS calls [19, 20], from the population in the given demand zone [21, 22], or from EMS interventions per 1,000 population and population structure [23]. The first way is possible if EMS statistics for all demand zones under consideration are available. The second way is a rough estimation that need not correlate with a real number of patients, since the demand for EMS is influenced by the population’s age structure that varies in a large-scale area, as we will demonstrate later on. The result is that the solution could not be optimal for real demand, and a so called surrogation error might arise [24]. Since historical data on ambulance interventions in every municipality were not available to us, we decided to predict EMS cases according to the third way, using a sample of patient data provided us by Falck Záchranná, a.s. and publicly available demographic data on population’s size and age structure. This way of demand-modelling results in a more realistic solution.

**Fig. 1:**
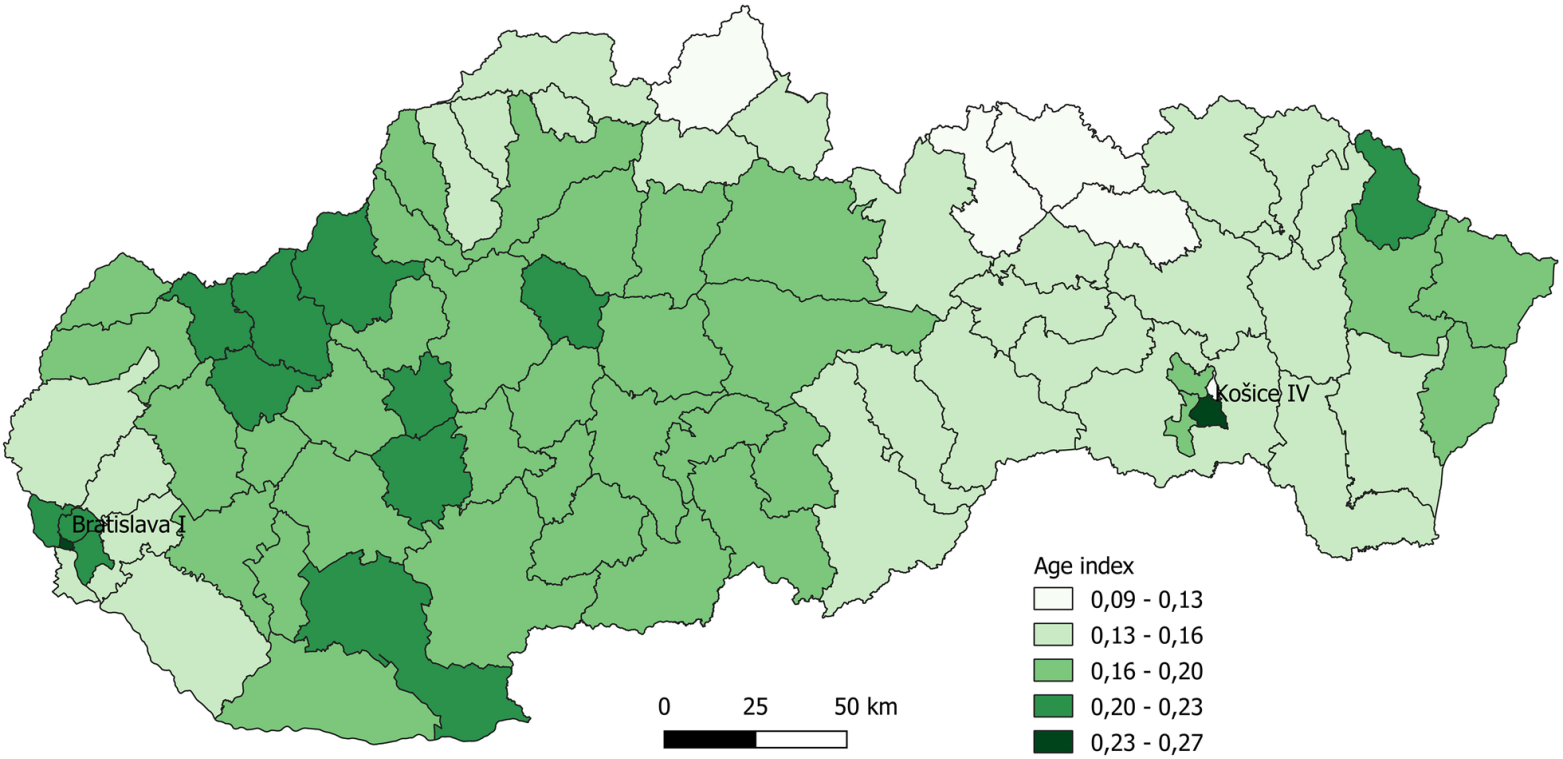
Spatial distribution of elderly people (Slovakia, 2015)

Falck Záchranná a.s. supplied us with depersonalized data on 149,474 patients served in the year 2015. Therefore demographic data we used for demand estimation are also for 2015. The 2015 population data published by the Statistical Office of the Slovak Republic reveal that people aged 65 years and over constitute 14.45% of the population. However, the population’s age structure is not homogenous throughout the state. To get a better idea about the age of people in different regions of Slovakia, we calculate an aging index for each territorial unit as the ratio of inhabitants who are at least 65 years old over inhabitants below the age of 65. The index varies a lot among municipalities (min = 0.012, max = 1.333, median = 0.177, mean = 0.189, sd = 0.083). At the district level the differences are not so conspicuous (min = 0.096, max = 0.263, median = 0.171, mean = 0.171, sd = 0.030) but their graphical presentation is more readable, and it illustrates the distribution of elderly people across the country (Fig. 1). The regions in the north with low index have a high birth rate. The highest index is in the central part of two largest cities Bratislava and Košice, where elderly people are in majority.

To calculate the share of elderly people in emergency dispatches, we use the Falck sample data. This dataset contains information about the time and date of each incident, the patient’s age, the initial medical diagnosis, and time stamps of the whole EMS trip. The data suggest that patients aged 65 years and over required 42.34% of the interventions.

Combining Falck data with publicly available statistics reported by the National Dispatch Center [25] and the population statistics published by the Statistical Office of the Slovak Republic we can calculate the rates of emergency interventions for various age groups according to Eq. (1):

1 $${rate}_{k}=1000\frac{D\cdot {Falck}_{k}}{{Pop}_{k}\cdot {Falck}_{total}}$$

where *rate*_*k*_ is the 1-year number of emergency cases per 1,000 persons in age group *k*, *Falck*_*k*_ is the number of patients in age group *k* in the Falck dataset, *Falck*_*total*_ is the total number of patients in the dataset, *D* is the total number of ambulance dispatches reported by the National Dispatch Center for the year 2015, and *Pop*_*k*_ is the number of inhabitants in age group *k*.

Within each age group we can further distinguish two groups of patients according to their initial medical diagnoses. The most severe diagnoses are denoted as the First Hour Quintet (FHQ), and they include: chest pain, severe trauma, stroke, severe respiratory difficulties, and cardiac arrest. Although the international definition of FHQ does not list unconsciousness, it is also a life-threatening condition. Therefore, after a consultation with emergency physicians, we decided to include it in FHQ. The FHQ conditions require immediate rescuing. If a call is recognized as a FHQ call, it gets the highest priority because every minute of delay in the response reduces patient’s chance of survival. The FHQ patients account for 26.51% of all patients in the Falck dataset.

**Fig. 2:**
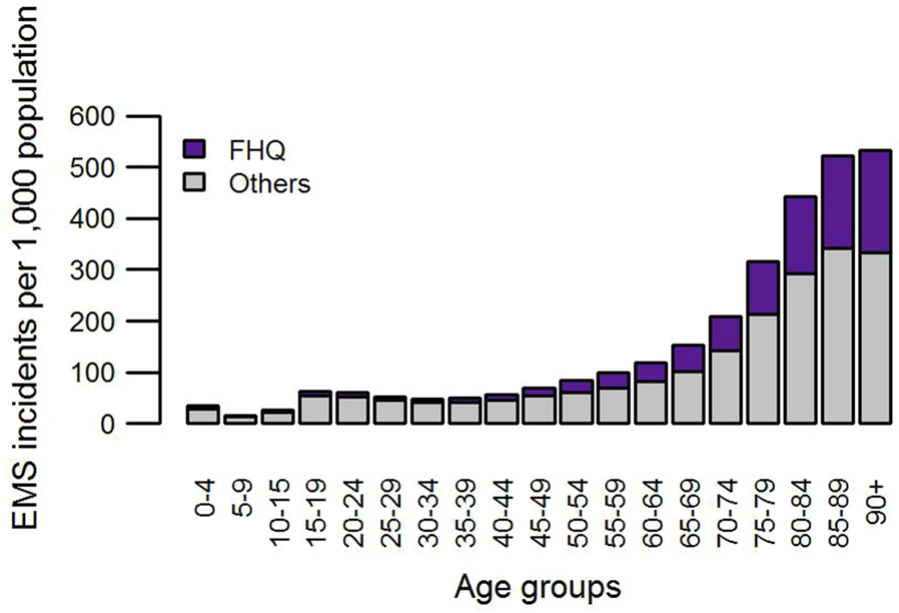
Emergency incident rates increase with age (Slovakia, 2015)

The analysis of EMS data reveals that the overall rates as well as FHQ rates increase with age (Fig. 2). The Spearman correlation is *ρ* = 0.95 for overall rate and *ρ* = 0.96 for FHQ rate. The dependency curve has an exponential shape, with the acceleration from the age of 65 years.

For the modelling purposes we will distinguish three age categories: (i) children in the age of 0–14 who have the lowest emergency incident rates (see Fig. 2), (ii) teens and nonelderly adults aged 15–64, and (iii) elderly people aged 65 years and over who call EMS the most frequently. The emergency incident rates for these categories are shown in Table 1. Based on the age structure and the rates we can estimate the annual number of EMS patients in municipality *j* according to Eq. (2):

2 $${b}_{j}=\sum _{k=1}^{3}{rate}_{k}{pop}_{kj}$$

where *rate*_*k*_ is the 1-year number of emergency cases per 1,000 persons in age group *k*, and *pop*_*kj*_ is the number of inhabitants in age group *k* in municipality *j*. Similarly, the annual number of high-risk patients ${b}_{j}^{FHQ}$ can be calculated using the rates of FHQ incidents.

**Table 1:** Emergency incident rates

Age group	Rate
0–14	26.09
15–64	69.12
65 +	267.07

### Modelling candidate locations and travel times

Candidate locations where stations can be placed are all municipalities and other villages that do not have local government but are the seats of stations today. There are 2,934 candidate locations in Slovakia and 2,928 of them are municipalities. The towns, boroughs and villages are represented by the nodes on the road network that are closest to the centre of the municipality. This way the calculation of travel times can be based on real network distances. The digital road network was downloaded from the OpenStreetMap database [26], which is a freely available source of geographical data. The travel times are related to deterministic speed of vehicles that depends on the quality of the road, its location inside or outside built-up area, the type of the movement (whether the ambulance drives at standard, or all possible speed with lights and sirens), and traffic volume that is higher in morning rush hours (from 6:30 to 9 am), as well as in evening rush hours (from 3 to 6 pm). The average ambulance speeds with regard to the road category and day time were derived from GPS records of ambulance trips by the Falck company (Tables 2 and 3) [21].

**Table 2:** Average speed in urban areas (kilometres per hour)

Road category	Lights and sirens			Standard speed		
	Speed	Morning rush hours	Evening rush hours	Speed	Morning rush hours	Evening rush hours
Motorway	100	92	90	90	86	86
Expressway	100	92	89	90	84	82
Important national road	65	60	56	46	42	40
National road	60	51	55	40	39	38
Local road	59	50	50	36	35	35
Residential road	8	6	4	8	7	7
Minor road	5	4	4	5	5	5

**Table 3:** Average speed in rural areas (kilometres per hour)

Road category	Lights and sirens			Standard speed		
	Speed	Morning rush hours	Evening rush hours	Speed	Morning rush hours	Evening rush hours
Motorway	110	100	102	100	100	100
Expressway	110	98	98	100	90	90
Important national road	101	88	89	86	67	65
National road	91	78	80	67	58	57
Local road	68	59	60	58	53	55
Minor road	5	5	5	5	5	5

### The bi-criteria mathematical programming model with coverage and response time objectives

The first model we propose is a bi-criteria model to maximize the expected coverage of high-priority FHQ patients and to minimize response time to all potential patients. The optimization procedure consists of the following steps:

1. Specify the stations that can be relocated.
2. Estimate the workload of ambulances using computer simulation.
3. Optimize the locations of the stations.
4. Specify geographic coordinates and assign ambulance types to the relocated stations.
5. If the distribution of the stations did not change, then stop. Otherwise, go back to step 2.

In the following text we describe the individual steps in detail.

To get a realistic solution, we do not allow all stations to change their current position. To decide which stations must remain where they are we apply two assumptions. Firstly, we suppose that ambulances in large towns are fully engaged. Therefore if the expected number of patients in a town exceeds the capacity of all ambulances currently stationed there, we do not allow them to change their positions. They are denoted as fixed and are not subject to the optimization. The demand volume in the corresponding municipalities is reduced by the total number of patients served by fixed stations. Secondly, we respect previous managerial decisions about multiple stations in a town where the estimated number of patients is less than the capacity of a station. There may be reasons that are not apparent to us but verified in practical operation. In such a case the model leaves one of the stations in the place and seeks better locations of the other stations. The preserved stations are not fully engaged by local residents in this case. Therefore the mathematical model fixes their positions but allows other municipalities to be assigned to their service area. The side effect of this pre-processing step is that the number of decision variables gets smaller and the complexity of the model is reduced.

A demand point is covered if an ambulance reaches it in a time standard. The desired service standard was set with regard to critical patients who are in life-threatening conditions and every minute delay in response time dramatically worsens their outcomes. These patients should be reached within 8 min, which is a widely accepted standard in most European countries for critical patients [27]. Thus assuming one minute pre-trip delay, we set the travelling time limit ${T}^{max}$ to the value of 7 min. Using this time standard we define the neighbourhood of a municipality. The neighbourhood consists of all candidate locations which are at most ${T}^{max}$ minutes far away.

To formalize the model, we introduce the following notation.

### Sets and indices

- *I*—set of candidate locations
- *I*_1_—set of fixed candidate location, where the ambulances are not fully engaged
- *J*—set of demand points (all municipalities)
- *i* ∈ *I*—candidate location
- *j* ∈ *J*—demand point
- *k*—index corresponding to the number of stations
- ${N}_{j}=\left\{i\in I:{t}_{ij}\le {T}^{max}\right\}$—set of candidate locations in the neighbourhood of demand point *j*

### Parameters

- *p*—number of stations to be sited
- *q*_*j*_—probability of an ambulance in the neighbourhood of demand point *j* being unavailable
- ${T}^{max}$—the desired service standard; ${T}^{max}=7$
- *b*_*j*_—the annual number of EMS patients in municipality *j* reduced by the capacity of the fixed stations
- ${b}_{j}^{FHQ}$—the annual number of FHQ patients in municipality *j*
- *t*_*ij*_ —shortest travel time from candidate location *i* to demand point *j*
- *st*_*i*_*—*the number of fixed stations at candidate location *i*
- ${n}_{j}=\left|{N}_{j}\right|$—the number of candidate locations in the neighbourhood of demand point *j*.

### Decision variables

$$x_{i}=\left\{ {\begin{aligned} {1,} &\quad{{\text{if a station is located at site }}i} \\ {0,} & \quad{{\text{otherwise}}} \\ \end{aligned} } \right.$$

$$y_{jk}=\left\{ {\begin{aligned} {1,} & \quad {{\text{if demand point }}j{\text{ is covered by at least }}k{\text{ stations}}} \\ {0,} & \quad {{\text{otherwise}}} \\ \end{aligned} } \right.$$

$$z_{{ij}}=\left\{ {\begin{aligned} {1,} & \quad {{\text{if demand point }}j{\text{ is served by the station located at site }}i} \\ {0,} &\quad {{\text{otherwise}}} \\ \end{aligned} } \right.$$

The following model is a mathematical programming formulation of the bi-criteria MEXCLP-*p*MP location model.

3 $$Maximize{\text{ }}f = \sum\limits_{{j \in J}} {\sum\limits_{{k = 1}}^{{n_{j} }} {b_{j}^{{FHQ}} } } \left( {1 - q_{j} } \right)q_{j}^{{k - 1}} y_{{jk}}$$

4 $$Minimize\,g = \sum\limits_{{i \in I}} {\sum\limits_{{j \in J}} {t_{{ij}} } } b_{j} z_{{ij}}$$

subject to

5 $$\sum\limits_{{i \in N_{j} }} {\left( {x_{i} + st_{i} } \right)} \ge \sum\limits_{{k = 1}}^{{n_{j} }} {y_{{jk}} } \quad for\,j\, \in \,J$$

6 $$\sum\limits_{{i \in I}} {z_{{ij}} } = 1 \quad for\, j \, \in \, J$$

7 $$z_{{ij}} \le x_{i} \quad for\,i\,\in I - I_1,\,j\,\in \,J$$

8 $$z_{{ij}} \le 1 \quad for\,i \in\,I_1,\,j\,\in\,J$$

9 $$\sum\limits_{{i \in I}} {\left( {x_{i} + st_{i} } \right)} = p$$

10 $$x_{i} \in \left\{ {{{0}},{{1}}} \right\}\,\quad for\,i\, \in \,I - I_1,\,j\, \in \,J$$

11 $$y_{{jk}} \in \left\{ {{{0}},{{1}}} \right\} \quad for\, j \in J,k = 1, \ldots ,n_{j}$$

12 $$z_{{ij}} \in \left\{ {{{0}},{{1}}} \right\} \quad for\,i\, \in \,I,\,j\,\in \,J$$

The objective function (3) maximizes the expected coverage of critical patients taking into account possible unavailability of ambulances. The term ${b}_{j}^{FHQ}\left(1-{q}_{j}\right){q}_{j}^{k-1}$ represents the increase in expected coverage of municipality *j* brought about by *k*th station. According to Eq. (5), sitting multiple stations in the neighbourhood of municipality *j* enables multiple variables *y*_*jk*_ take the value of one and account for the increase in coverage. The objective function (4) minimizes the total travel time needed by the ambulances to reach all patients. The average travel time is equal to the total travel time divided by the number of all patients. Constraints (6) assign every municipality *j* to the service area of exactly one station *i*. Constraints (7) ensure that if a municipality *j* is assigned to a node *i*, then a station will be opened at the node *i*. Constraints (8) allow a municipality *j* to be served from a fixed station that is not fully engaged. Constraint (9) limits the number of located stations to their current amount. The obligatory constraints (10)–(12) specify the definition domains of the variables.

To solve the bi-criteria model, we used the lexicographic method. The method assumes a ranking of the objective functions according to their importance but in contrast to scalarizing and goal programming approaches, it does not require additional parameters. It is an iterative method. In the first step, the problem is optimized with the most important objective. If it has the only optimal solution, then this solution is also the best solution to the original multiple criteria problem and the method finishes. Otherwise, the problem with the second most important objective function is solved subject to a condition that the first objective function value will not worsen. The process repeats until a single optimal solution is found. In our problem, we consider the expected coverage of high-priority patients to be more important than the average response time. First, the single criterion model (3), (5), (9)–(11) is solved. The model maximizes the expected coverage of high-priority patients. Let us denote its optimal objective value as ${f}^{*}$. Then the weighted *p*-median problem (4), (6)–(12) with additional constraint (13) is solved.

13 $$\mathop \sum \limits_{{j \in J}} \mathop \sum \limits_{{k = 1}}^{{n_{j} }} b_{j}^{{FHQ}} \left( {1 - q_{j} } \right)q_{j}^{{k - 1}} y_{{jk}} \ge f^{*}$$

Constraint (13) assures that the expected coverage of most critical patients will not worsen when minimising the average response time for all patients.

The structure of the MEXCLP model (3), (5), (9)–(11) makes it easy to solve by a general-purpose solver. The weighted *p*-median problem (4), (6)–(10), (12), (13) is an *NP*-hard problem with a huge amount of variables that cannot be solved exactly. Instead, an approximation algorithm has to be used. We chose the kernel search method and adjusted it to our specific problem. Kernel search is a recently developed matheuristic that has been successfully applied for solving mixed integer linear problems (MILPs) with binary variables [28, 29]. In principle, it is a decomposition method that in sequence solves sub-problems of the original MILP problem. A sub-problem consists of a subset of decision variables. The subset contains only promising variables (a kernel) and a small subset of the remaining variables. The sub-problems are solved using a general-purpose MILP solver as a black-box. Thus the method benefits from the efficiency of the state-of-the-art solvers.

The solution of the model defines the municipalities where the stations will be deployed (at most one station in a municipality). This output is merged with the pre-processed fixed locations, resulting in multiple stations in more populated towns and boroughs. However, at this moment we do not have specific addresses, but multiple stations are regarded as located in the single (central) node of the municipality. The geographic positions of the stations inside a given municipality are determined afterwards, using a rule-of-thumb. We proceed from the existing locations. The addresses of fixed stations are preserved. A new station, if there is one, is placed at the municipality’s central node on the road network. If one or more stations out of multiple existing stations are removed, they are selected randomly*.*

The model does not distinguish the types of emergency units. However, their distribution, especially the locations of ALS ambulances affect the efficiency of the system because an ALS ambulance is always dispatched to the high-priority call. We distribute ambulances among the optimized station locations a-posteriori in the following way: first, we retain the type of fixed stations that are disregarded in the optimization, and also the type of those stations whose positions were not changed by the optimization model. As regards the relocated stations, firstly we place ALS ambulances close to their current positions that are mainly in hospitals. The remaining stations are assigned by BLS ambulances.

The probability of an ambulance in the neighbourhood of a municipality being unavailable is an input parameter to the bi-criteria model. It is estimated using computer simulation of the EMS system. The probability is calculated as the average workload of potential ambulances in the neighbourhood. However, the workload depends on the distribution of the ambulances and therefore it is de facto the output of the model. Since we need it as input, it must be estimated a-priori. Initially, the workload is estimated using the current station location. If there is at least one ambulance currently operating in a candidate location, then the probability of this candidate is calculated as the average workload of currently operating ambulances. If the candidate does not have a station today, then its workload is set to the average workload of the stations that are in the 30 min neighbourhood of the candidate. The optimized distribution of the stations is submitted to simulation to obtain workload for the second run of the model. The process is repeated until convergence is achieved. Convergence is measured by ambulance distribution. When the locations in two successive solutions are (almost) identical, then the process stops.

### The hierarchical model minimizing response time

To cope with the two-tiered EMS system that works in Slovakia and in many other countries, it is desirable to design an optimization model where different types of EMS units are taken into account. The EMS system with two vehicle types can be viewed as a hierarchical facility system. Using the classification by Şahin and Süral [30], we face a multi-flow, nested, and non-coherent system. If the objective is to minimize the total distance (or travel time, respectively) from demand zones to the closest ALS and BLS stations, then the hierarchical *pq*-median problem is to be solved. We propose a modification of the *pq*-median model by Serra and ReVelle [31]. Serra and ReVelle focus on coherent systems where all demand areas assigned to a lower level facility must be assigned to one and the same upper level facility. However, this condition does not hold in the EMS system we deal with. Thus we have amended their model for a non-coherent system. Moreover, we allow an upper level facility to be located only at a site where a lower level facility is opened.

In addition to location variables *x*_*i*_ that decide on location of stations regardless of their type, we need another set of variables that model the decisions on locating only ALS stations:

$$u_{i}=\left\{ {\begin{aligned} {{{1,}}} &\quad{{\text{if an ALS station is located at site }}i} \\ {0,} & \quad {{\text{otherwise}}} \\ \end{aligned} } \right.$$

Service areas of the ALS stations are modelled using the following allocation variables:

$$v_{{ij}}=\left\{ {\begin{aligned} {1,} &\quad {{\text{if demand point }}j{\text{ is served by the ALS station located at site }}i} \\ {0,} &\quad {{\text{otherwise}}} \\ \end{aligned} } \right.$$

The lower level of the hierarchical *pMP* model consists of the objective function (4) and constraints (6)–(10) and (12). It decides on location of stations regardless of their type and creates their service areas. The upper level of the model decides which stations opened in the lower level will house ALS ambulances:

14 $$Minimize\sum _{i\in I}\sum _{j\in J}{t}_{ij}{b}_{j}{v}_{ij}$$

subject to

15 $$\sum\limits_{{i \in I}} {v_{{ij}} } = 1\, \quad for\, j \in J$$

16 $$v_{{ij}} \le u_{i} \, \quad for \,i \in I,\,j \in J$$

17 $$u_{i} \le st_{i} + x_{i} \,\quad for\,i\, \in I$$

18 $$\sum _{i\in I}{u}_{i}=r$$

19 $$u_{i} \in \left\{ {0,1} \right\}\,\quad for \,i \in I$$

20 $$v_{{ij}} \in \left\{ {{{0}},{{1}}} \right\}\,\quad for \,i \in I,\,j \in J$$

The objective function (14) minimizes the total travel time needed by the ALS ambulances to reach all patients. Constraints (15) assign every municipality *j* to the service area of exactly one ALS station *i*. Constraints (16) say that a municipality *j* can be assigned only to an open ALS station. Constraints (17) allow an ALS ambulance to be allocated only to a station opened at the lower level of hierarchy. Constraint (18) limits the number of located ALS stations to their current amount *r*. The remaining constraints (19) and (20) specify binary variables.

Both levels of the hierarchical model can be solve exactly using an efficient method by Janáček and Kvet [32]. The ALS ambulances will be allocated to those fixed or relocated stations, for which *u*_*i*_ = 1 in the optimal solution. The remaining stations will house a BLS ambulance.

### Computer simulation model

A detailed computer simulation model was developed [21]. Its purpose in this study is twofold: (i) to estimate the workload of ambulances as the input for the mathematical programming model, and (ii) to evaluate the performance of the EMS system with the infrastructure proposed by the model. The computer simulation models the reality on a less abstract level than a mathematical programming model does, therefore it provides us with better idea of the performance of the projected system. It calculates such quantitative indicators that cannot be derived from a mathematical programming model itself.

We implemented a self-driven, agent-based simulation model using AnyLogic simulation software. The model is developed on the Java simulation core. We implemented a library of classes and functions in Java for the simulation support.

The model was calibrated using the data sources as follow:

1. Publicly available statistics published by the National Dispatch Center;
2. The positions of the stations provided by the Ministry of Health;
3. A sample of patient data provided by Falck Záchranná a.s.;
4. LandScan data on population distribution;
5. OpenStreetMap data on the road network;
6. Historical data on the average ambulance speed with regard to the road category and day time provided by Falck Záchranná a.s.

The dataset obtained from Falck Záchranná a.s. allows us to extract important knowledge. First of all, the time distribution of calls can be revealed. With regard to the seasons and weekdays, we did not observe statistically significant differences in the number of calls. However, the call rates change significantly during a day. We can observe two peaks, one between 9 and 11 am and the other one between 5 and 9 pm. So the arrival of calls is modelled as a non-homogeneous Poisson process with the arrival rate varying depending on the time of day.

The spatial distribution of patients is modelled using the LandScan database [33]. LandScan data represent an ambient population (average presence of people over 24 h). A grid cell corresponds to an area of 30′′ × 30′′ (arc-seconds) in the WGS84 geographical coordinate system. The territory of the Slovak Republic is covered by 70,324 grid elements. The call that has been generated by the Poisson process is assigned to a grid cell with a probability that is proportional to its population. Inside the grid cell, the call is assigned randomly to a node on the road network.

The model captures all important processes presented in the management of emergency patients including precise modelling of the distribution of processing times.

The main features are the following:

- As to demand modelling, we take into account three important characteristics: the arrival distribution, the geographical distribution and the priority of calls.
- The model of the service time comprises all phases of the ambulance trip—the journey to a patient, treatment of the patient at the site of the incident, transportation to a hospital, drop-off time in the hospital, and the journey back to the base station.
- The movement of an ambulance respects the underlying road network.
- The on-scene time is modelled using a probability distribution that depends on the patient’s diagnosis and crew’s qualification.
- The probability of the transportation of a patient to the hospital depends on the type of the intervening crew. The real data show that 77% of the patients treated by a paramedic team and 51% of the patients treated by a physician are transported to a hospital. If a patient has to be transported to a hospital, then the closest hospital complying with their condition and age is chosen (for example, there are hospitals specialized in cardiovascular diseases or children’s hospitals).
- In the hospital, the rescue team hand over the patient to the hospital staff, then they may spend some time cleaning and resupplying the vehicle. The time needed to perform these tasks is called drop-off time. The probability distribution of the drop-off time is modelled separately for every hospital. In most cases, the Erlang distribution fits well. The average drop-off time ranges from 7.1 to 36.2 min.
- After leaving the hospital, the ambulance is available to respond to another call. It means that the ambulance can be dispatched to another call on its way home. The logic of ambulance dispatching approximates very well the rules adopted in practice. For example, an ambulance can be dispatched to another rescue while it is returning to its home station. In the simulation model it means that it is possible to change the destination of the ambulance while it is moving along the road.
- Secondary transports are modelled as well, since they reduce the availability of the ambulances. A secondary transport is a planned activity where an ambulance does not respond to an emergency call but transports a patient or medical material between two hospitals.

These features represent a significant improvement in comparison to other simulation models reported in the literature.

The model was verified using the following techniques recommended also in [12]:

- Animation to graphically visualize the movements of vehicles through the road network to check whether the rescue process and the chosen routes are as expected. During the rescue process, the colour of the vehicle changes to reflect its current state (movement to a patient, stay at the scene, transport of the patient to a hospital, return back to the base station).
- Face validity by consulting EMS specialists who evaluated the model’s conception and output behaviour compared to the real-world system.
- Traces to track the movement of vehicles and occurrence of every event in the model (call arrival, vehicle assignment, destination hospital selection etc.) so as to validate the correctness of the model logic.
- Sensitivity analysis by performing a comprehensive set of simulation experiments with different values of input parameters (e.g. arrival rate or hospitals with emergency departments) to determine if the model’s output is as expected.

The output of the simulation model includes the following EMS performance indicators:

1. Average response time, since it has been monitored by the National Dispatch Center;
2. Percentage of calls responded to within 15 min, because a 15-min response time is regarded as standard in Slovakia;
3. Number of municipalities with the average response time longer than 15 min;
4. Average response time for the high-priority (FHQ) calls and the percentage of these calls responded to within 8 min;
5. Average ambulance workload and its variation.

## Results

The mathematical models were solved using the solver Gurobi Optimizer 8.1.1. The exact method [32] for the *p*-median problem is very efficient. The computing time was 580 s for the weighted *p*-median model and 607 s for the hierarchical *pq*-median model, respectively. The kernel search method for the MEXCLP-*p*MP model was implemented in Java language. A single run of the model for one workload setting took on average about 6 min. Altogether 4 iterations of the model were needed until convergence in the station distribution was achieved.

The simulation model, described above, was used to evaluate the current locations of emergency stations, as well as the optimized locations proposed by mathematical models.

The results of the simulation experiments are summarised in Table 4. The simulation experiment for one set of station locations consisted of 10 replications. One replication simulated 91 days of EMS performance. For response times, the mean values from 10 replications and 95% confidence intervals are reported. For coverage indicators, the mean values are given. The best values of the indicators are displayed in bold. Although the ambulance trip data and the population data are related to the year 2015, we validation of the model was performed in 2017 using the latest positions of the stations (5 stations shifted in the meantime). That is why we refer June 2017 as the current date.

**Table 4:** Performance indicators for the current and optimized locations

Indicator	Current locations (June 2017)	MEXCLP-pMP	pMP	Hierarchical pMP
Response time for all patients (min)	11.52 (11.50; 11.54)	10.75 (10.73; 10.77)	10.56 (10.55; 10.57)	**10.55** (10.52; 10.58)
% of calls responded to within 15 min	75.26	79.97	80.28	**80.33**
Number of municipalities with the average response time longer than 15 min	868	676	**600**	601
Response time for high-priority patients (min)	11.37 (11.34; 11.40)	10.62 (10.59; 10.65)	10.48 (10.45; 10.52)	**10.44** (10.40; 10.49)
% of high-priority calls responded to within 8 min	38.84	43.75	44.23	**44.36**
Average ambulance workload (%)	31.98	31.89	31.84	**31.78**
Coefficient of variation of ambulance workload	0.29	**0.24**	**0.24**	**0.24**

**Fig. 3:**
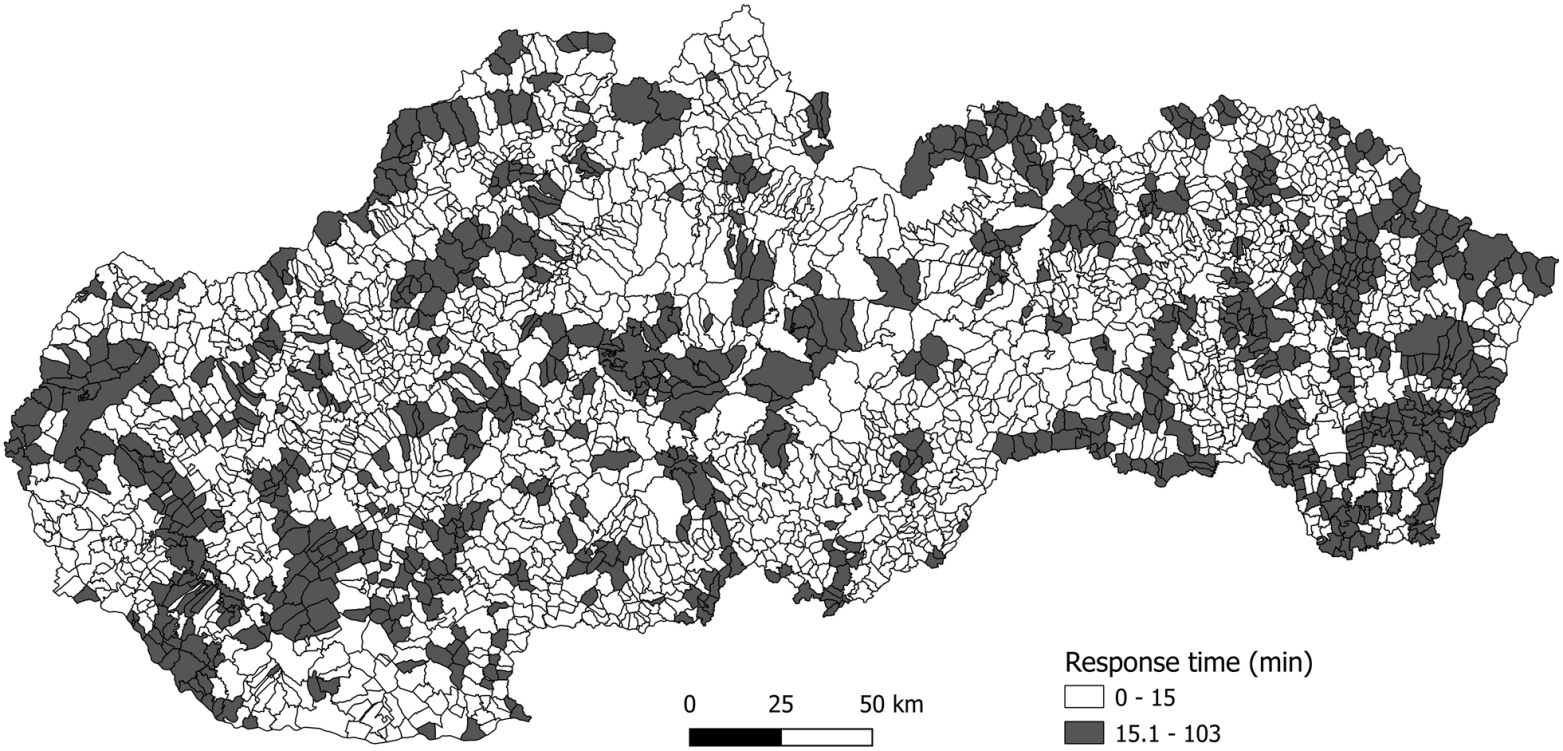
Municipalities with the average response time longer than 15 min, current station location

The computer simulation of the current (2017) system revealed that the system is short of the target to reach 95% of patients within 15 min. The real accessibility within this time limit is only 75.26%. 868 municipalities (almost 30%) have the average response time longer than 15 min (Fig. 3). The Slovak system also exhibits poor performance regarding the 8-min response-time standard for the high-priority calls. Only 38.84% of critical patients are reached within 8 min, which is far less than the EU average of 66.9% [27]. The average ambulance workload is 31.98%, which corresponds to other EMS systems worldwide where ambulances are typically busy at least 30% of the time [19].

From the rest of the table we can observe that the reorganization of the system has a positive effect on the performance. Both MEXCLP-*p*MP and *p*MP models relocate approximately 78% of the stations (150 and 151, respectively). Regardless of the ambulance allocation, the mathematical programming models reduce significantly the overall average response time, as well as response time for the most critical patients (their confidence intervals do not overlap with the confidence intervals for status quo). In parallel with reducing the response time, the accessibility within a given time threshold is increasing.

As regards the two policies of allocation of ALS ambulances, the hierarchical model that incorporates the decisions on particular ambulance types achieves better results than the models where the type of the stations is defined in a post-optimization process. The most important improvement is in the accessibility of the critical patients. In comparison to the current state, the average response time of them is reduced by 56 s. It may seem that one minute is not too much, but one has to realize that for a person who is in a life-threatening condition, such as a cardiac arrest, the line between life and death is very thin, and every second matters. Cardiac arrest and unconsciousness are the most frequent diagnoses of those patients who die before or during the rescue operation. From the Falck sample data on these patients we can derive the survival probability as a function of response time *t*. The survival probability function is as follows [21]:

21 $$s\left( t \right) = \frac{1}{{1 + \exp \left( { - 2.04492 + 0.045427t} \right)}}$$

From the sample data and the total number of patients reported by the National Dispatch Center we can estimate that in 2019 there were 26,003 most-critical patients in Slovakia. The reduction of the average response time by 56 s means that the survival probability increases by 0.61%. As a result, by 142 more patients could be saved. We think this is a significant improvement since every life matters.

**Fig. 4:**
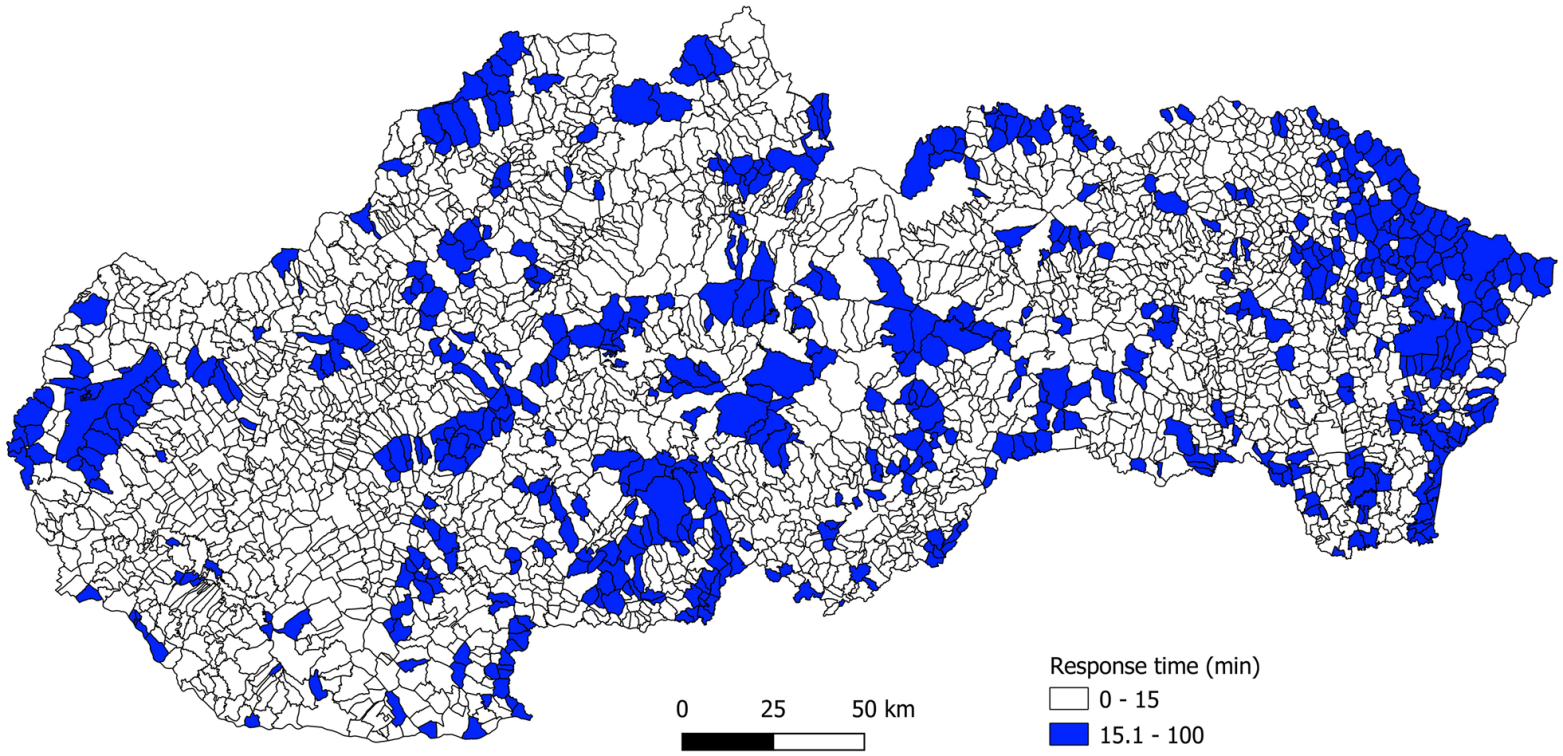
Municipalities with the average response time longer than 15 min after optimization by the hierarchical *p*MP model

Regardless of the model and ALS allocation policy, relocating the stations improves the accessibility mainly in the densely populated western part of the country. The most successful hierarchical *p*MP model reduces the overall number of municipalities with the average response time greater than 15 min by 267 (31%) (Fig. 4). The model also generates the smallest ambulance workload and thus increases the probability that the closest ambulance will be available when needed. At the same time, ambulance workload is distributed more evenly (coefficient of variation is less than at present).

## Discussion

Our study is the first to compare different objectives for location of EMS stations in a large urban–rural area. This way it fills the gap in ambulance location literature since the research so far has been concentrating on urban rather than rural or mixed areas [34]. Modelling the EMS system for a heterogeneous urban–rural area is more challenging than it is for a homogeneous region. There may be different time standards for densely and rarely populated areas, there are big differences in ambulance workload, different road network quality, traffic volume, and the distance to the nearest hospital. Therefore the results of urban-oriented studies cannot be directly applied to a region with diverse topography and demography.

We concentrate on two objectives that are supposed to mostly influence the outcomes of emergency medical services, particularly the maximum coverage and the minimum average response time. Previous studies [15, 35, 36] suggested that the maximum coverage objective itself does not perform well. The response time related objectives such as the average response time [35, 36] and maximum survivability [15], result in a better system performance than the maximum coverage objective. Although these outcomes were derived for metropolitan areas, we considered them to be a good starting point for our research. Another viewpoint is that the coverage ensures equity among the patients, at least to some extent. That is why we have developed a multi-criteria model that combines the maximum coverage of high-risk patients with the minimum average response time to all patients. This model resolves the deployment of ambulance stations. Ambulances are assigned to the open stations under a heuristic rule. The other model we have proposed is a hierarchical model that minimizes the average response time to all patients with both types of emergency units. Finally, the simulation study has revealed that the model with the coverage objective improves the existing system but it is outperformed by models that rely on the response time only. Our findings are in compliance with Felder end Brinkmann [37] who give theoretical evidence that an equal access approach to the EMS provision does not maximize the number of lives saved.

A reason for poorer performance of the coverage objective may be in the imprecise estimation of busy fractions of ambulances. Busy fractions of the candidate locations that do not have a station today are set at the average value of the stations in the neighbourhood. It might be a value too optimistic for many candidate locations. Covering models in general do not differentiate different locations within the same response time threshold. Therefore, what may happen is that the model decides to place some stations in small villages near large towns. From the point of view of the covering objective, all patients in the town are considered perfectly satisfied with the service. However, the real workload of ambulances would be enormous. For example, let the village, whose demand is 1, be 5 min away from the town with the call volume of 100. If the ambulance was located in the village, travelling to the scene would add 500 min to its workload compared to 5 min, if the station was located in the town. So we do not recommend using coverage criteria for a large-scale region.

In our previous studies we experimented with several other models using response time objectives. The maximum response time was examined in [21]. The capacitated version of the *p*-median problem was investigated in [29]. Here the average response time was minimized, provided the number of calls an ambulance could serve was restricted. None of these models outperformed the hierarchical *pq*-median model.

The important part of the overall emergency call-to-care interval is the patient access time interval (PATI) measured from EMS vehicle arrival at the incident site to the time EMS personnel contact the patient. This time interval accounts for 10 to 44% of the overall emergency call-to-care interval [38]. Although reducing PATI may improve patient outcomes, it is not affected by the locations of ambulance base stations. Rather community- specific strategies are to be developed to overcome the patient-access barriers. In our study, PATI is modelled in computer simulation as a part of the on-scene time.

The results of our study can be applied in all countries with a tiered EMS system that utilizes different types of emergency units, dispatching ALS units to the most severe events and using BLS units for non-urgent and scheduled transport of stable patients. Tiered systems apply the Franco-German model of EMS delivery where the crew is qualified to treat patients in their homes or at the scene. Such systems are common in many European countries such as Germany, France, Greece, Austria, Czech Republic, Hungary, and Poland [39–41].

If the results of our study were to be used to reorganize an existing system, we recommend to assess the current system thoroughly. None of the EMS systems in the countries mentioned above is a greenfield project. There may be a lot of decisions and measurements that already work effectively, and the reorganization should respect them. Here we have in mind especially the decisions on which stations should remain in their current positions. Another reason for this pre-processing step is that the *p*-median model does not allow multiple stations to be opened at one site. This is the main drawback of the proposed approach.

Another limitation of our approach is the absence of investment costs connected with the relocation of the stations. A limited budget is always to be taken into account in real life. Together with the resistance of professionals and public to changes, it may lead to a limited number of relocated stations. Nevertheless, our models are able to cope with such a restriction via *p* and *r* parameters.

Furthermore, we would like to emphasize the necessity of demands being modelled carefully. The changes we propose are based on the current demand distribution. Even though the impact of the age structure has been considered, we do encourage a serious analysis of the demography and morbidity trends in the given region to be conducted.

Finally, we recommend the usage of computer simulation as a validation tool. The simulation model itself is not able to suggest the best station locations, however, it is useful in evaluating various scenarios that include not only the number and distribution of the EMS stations but also such factors as the types of ambulances, destination hospitals, or dispatching policies. To get a credible output, the model must capture all processes on the emergency care pathway including reliable distributions of processing times. In the future, we will elaborate demographic prognoses for particular regions of the country and incorporate them into the model. Then the simulation will allow us to predict the future performance of the EMS system, and to identify the resources necessary for ensuring a satisfactory quality of emergency care.

## Conclusions

In this paper we present the utilization of different operations research techniques to support the decision making process regarding placing the EMS stations over a large urban–rural area. A bi-criteria mathematical programming model is proposed. The criteria include the coverage of high-priority patients and response time in relation to all patients. The model is compared to the *p*-median model with a single response time objective and to a hierarchical *pq*-median model that involves two types of emergency units. The following conclusions can be derived from our empirical study:

1. All mathematical models make EMS performance better than the current status is by relocating some stations.
2. The minimum average response time objective produces better results than the maximum coverage objective.

## Data Availability

The datasets used and/or analysed during the current study are available from the corresponding author on reasonable request.

## Abbreviations

ALS: Advanced life support
BLS: Basic life support
EMS: Emergency medical service
FHQ: First hour quintet
MEXCLP: Maximum expected coverage location problem
MILP: Mixed integer linear problem
MLCP: Maximal covering location problem
PATI: Patient access time interval
pMP: *p*-Median problem
